# Glucose Variability and *β*- Cell Response by GLP-1 Analogue added-on CSII for Patients with Poorly Controlled Type 2 Diabetes

**DOI:** 10.1038/srep16968

**Published:** 2015-11-26

**Authors:** Chia-Hung Lin, Sheng-Hwu Hsieh, Jui-Hung Sun, Jir-Shiong Tsai, Yu-Yao Huang

**Affiliations:** 1 Division of Endocrinology and Metabolism, Department of Internal Medicine, Chang Gung Memorial Hospital, Linkou, Taiwan; 2 Graduate Institute of Clinical Medical Sciences, Chang Gung University, Taoyuan, Taiwan; 3 Sun Yat-Sen Cancer Center, Taipei, Taiwan

## Abstract

The effects of twice-daily GLP-1 analogue injections added on continuous subcutaneous insulin infusion (CSII) in patients with poorly controlled type 2 diabetes (T2DM) were unknown. After optimization of blood glucose in the first 3 days by CSII during hospitalization, patients with poorly controlled T2DM were randomized to receive CSII combined with injections of exenatide or placebo for another 3 days. A total of 51 patients (30 in exenatide and 21 in placebo groups) with mean A1C 11% were studied. There was no difference in mean glucose but a significant higher standard deviation of plasma glucose (SDPG) was found in the exenatide group (50.51 ± 2.43 vs. 41.49 ± 3.00 mg/dl, *p* = 0.027). The improvement of incremental area under the curve (AUC) of glucose and insulinogenic index (Insulin_0–peak_/ Glucose_0–peak_) in 75 g oral glucose tolerance test was prominent in the exenatide group (*p* < 0.01). The adiponectin level was significantly increased with exenatide added on (0.39 ± 0.32 vs. −1.62 ± 0.97 μg/mL, in exenatide and placebo groups, respectively, *p* = 0.045). In conclusion, the add-on of GLP-1 analogue to CSII increased glucose variability and the *β* - cell response in patients with poorly controlled T2DM.

In the poorly controlled patients with type 2 diabetes mellitus (DM), insulin therapy is the treatment of choice to control glucose levels on target. Actually, however, the general control rate is not good which is partially due to the complex etiology in type 2 DM. Glucagon-like peptide-1 (GLP-1) is secreted from enteroendocrine L cells of the intestinal mucosa and is released into the portal circulation in response to meal ingestion^1^ through posttranslational processing of proglucagon by prohormone convertase-1 in its secretary cells^2^. GLP-1 enhances insulin secretion and inhibits glucagon release in a glucose-dependent manner, prompting the development of GLP-1-based therapies for the treatment of diabetes^3^. GLP-1-based diabetes therapies affect glucose control through several mechanisms, including slowed gastric emptying, regulation of postprandial glucagon, reduction of food intake, and enhancement of glucose-dependent insulin secretion without the risk of hypoglycemia^4^. The combination with twice-daily exenatide has been shown to improve glycemic control in patients with type 2 diabetes that had been treated with basal-only insulin regimen^5^. But the effect of GLP-1 analogue on intensive insulin therapy for patients with type 2 DM remains unknown. To study the effect of GLP-1 analogue in insulinized type 2 DM patients, the first priority is to optimize insulin therapy. Continuous subcutaneous insulin infusion (CSII) or insulin pump is a viable choice for patients with type 1 or type 2 DM who want close-to-physiologic insulin treatment^6^. By means of the insulin pump therapy during hospitalization, we can optimize the sugar control profile efficiently^7^. We can further evaluate the clinical response under GLP-1 analogue precisely in these patients with poorly controlled type 2 DM.

## Results

### Clinical manifestations

There were 55 patients under screening and 4 patients were excluded because of the patients’ decision. Finally, fifty-one patients were randomized. The gender, mean age, body mass index (BMI), duration of diabetes mellitus, C-peptide and A1C levels were not different between the GLP-1 analogue and placebo groups (Table 1). The mean glucose values were lower in use of the GLP-1 analogue than placebo even though not statistically significant (143.93 ± 4.15 vs. 153.36 ± 5.13 mg/dl, *p* = 0.167) (Table 2). When comparing the glucose variation between two groups, the standard deviation of plasma glucose (SDPG) adjusted for baseline values was significantly higher in the GLP-1 analogue than placebo group (50.51 ± 2.43 vs. 41.49 ± 3.00 mg/dl, *p* = 0.027). The insulin dose did not show significant difference between two groups in both baseline and endpoint (Supplement Table 1).

**Figure 1:**
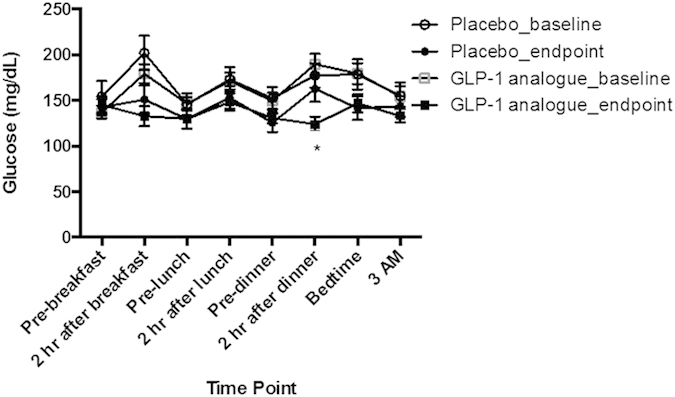
Baseline and end-of-study eight-point blood glucose profiles (mean ± SEM). ^*^p < 0.05 between GLP-1 analogue and placebo at endpoint by ANCOVA test after adjustment of adjustment of sex, mean age, BMI, duration of diabetes mellitus, C-peptide and A1C levels.

The 8-point blood glucose profiles were shown in Fig. 1. The glucose value at 2-hour after dinner was significantly lower in the GLP-1 analogue group than in the placebo group (*p* < 0.05) at the end of study by ANCOVA test after the adjustments of sex, mean age, BMI, duration of diabetes mellitus, C-peptide and A1C levels. Supplement Table 2 showed all adverse events that occurred in at least 3% of GLP-1 analogue recipients. More GLP-1 analogue recipients than placebo recipients had abdominal fullness (11 [36.7%] vs. 0 [0%], respectively; *p* = 0.001). No treatment-emergent pancreatitis or acute renal failure occurred. The frequency of hypoglycemia was not different between two groups among overall, nocturnal and severe categories.

### *β*- cell function and insulin resistance

Table 3 revealed the changes in *β*- cell function and insulin sensitivity measurements over the course of the study. Compared with the placebo group, the incremental glucose AUC and insulinogenic index (Insulin_0–peak_/Glucose_0–peak_) were significantly improved in the GLP-1 analogue group (*p* < 0.01). In addition, the *β*- cell response showed significant improvement in 2-hour glucose and glucose change (△ G (0–120)) during 75 g oral glucose tolerance test (OGTT) (*p* < 0.001, Supplement Figure 2). The insulin increment from 0 minute to 120 minutes (△ I (0–120)) showed also significant responsiveness in the GLP-1 analogue group during 75 g OGTT (*p* = 0.030, Supplement Figure 3). The Matsuda index for insulin sensitivity showed improvement from baseline to the end of the intervention but without significant differences between the two groups. The other established index of insulin secretion (C-peptide/glucose [ng. ml^−1^. mg^−1^. dl]) during 75 g OGTT^8^ showed significant improvement of *β*- cell function at 30, 60, 90 and 120 minutes (Supplement Table 3) in the GLP-1 analogue group compared with the placebo group.

**Figure 2:**
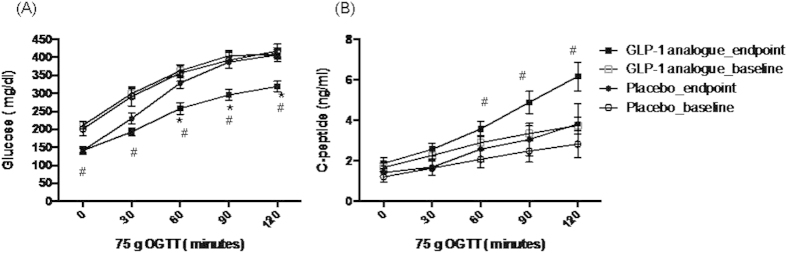
Mean ± SEM for plasma glucose (A) and C-peptide (B) concentration during the 75 g OGTT at baseline and endpoint in both groups. ^*^p < 0.05 between groups at end point. ^#^*p* < 0.05 endpoint vs. baseline at GLP-1 analogue group.

The plasma glucose and C-peptide excursions at each OGTT time point were illustrated in Fig. 2. The glucose values were significantly lower from baseline to endpoint at 0, 30, 60, 90, 120 minutes (*p* < 0.05) in the GLP-1 analogue group and lower than the placebo group at 60, 90, and 120 minutes at endpoint (*p* < 0.05). As for C-peptide values, the data was significant higher at endpoint than baseline at 60, 90, and 120 minutes (*p* < 0.05) in the GLP-1 analogue group. There was no significant change from baseline to endpoint at placebo group for both glucose and C-peptide levels.

### Effects of GLP-1 analogue on cardiovascular risk biomarkers

The adiponectin level was significantly increased from baseline to endpoint in the GLP-1 analogue group than in the placebo group (0.39 ± 0.32 vs. −1.62 ± 0.97 μg/mL, *p* = 0.045, Table 4). In addition, the hsCRP, BNP and 24-hour urine 8–iso-PGF_2α_ values were all decreased from baseline to endpoint in both groups but the differences were not statistically significant between the GLP-1 analogue and placebo recipients (−1.03 ± 0.81 vs. −0.61 ± 0.46 mg/L, *p* = 0.508; −8.68 ± 3.34 vs. −8.21 ± 4.65 pg/mL, *p* = 0.991 and −47.27 ± 58.39 vs. −186.04 ± 84.45 pg/mg /creatinine, *p* = 0.152, respectively).

In the GLP-1 analogue group, the correlation of changes of adiponectin level, insulinogenic index, and SDPG with baseline information of patients were further analyzed (Table 5). The change of adiponectin level was negatively correlated with the BMI and serum C-peptide level (Pearson correlation coefficients, −0.403 with *p* = 0.027 and −0.426 with *p* = 0.019, respectively).

## Discussion

This was the first prospective, randomized, placebo-controlled study related to the GLP-1 analogue add-on intensive insulin therapy with CSII in poorly controlled patients with type 2 DM. With characteristics of the GLP-1 analogue, exenatide, added to CSII in hospitalization, we could evaluate the real response of the GLP-1 analogue in patients with poorly controlled type 2 diabetes mellitus by optimizing the baseline glucose. The combination of exenatide and insulin has previously been evaluated in clinical trials^5^,^9^,^10^. In a placebo-controlled trial, exenatide added to insulin glargine reduced A1C by approximately 0.7%^5^. Another randomized trial examined the replacement of insulin with exenatide in patients with type 2 diabetes and found that glycemic control deteriorated in 38% (11 of 29) of the patients who received exenatide compared with 19% (3 of 16) of the patients who continued with insulin^11^. Patients who lost glycemic control were more likely to have a longer duration of disease, lower C-peptide concentrations (suggesting less endogenous *β*- cell function), and larger insulin requirements at baseline. However, the combined use of basal-bolus or CSII and exenatide could maintain minimal *β*- cell function and potentiate the clinical effect of exenatide.

The decreased glucose excursion postprandially has been reported in the combination of basal insulin with GLP-1 analogue^5^. In contrast, the higher SDPG in our study group at the endpoint demonstrated the impact of GLP-1 analogue on the postprandial glucose in even poorly controlled patients. The insulin dose did not show significant difference between the two groups. The hypoglycemia rate was also similar between GLP-1 analogue and placebo recipients. The corresponding increased adiponectin level could be coupled with a decrease of insulin resistance. Since the blood glucose has been normalized by CSII before GLP-1 analogue add-on, the exenatide induced postprandial glucose lowering effect combined with decreased insulin resistance could cause the elevation of SDPG compared to the placebo. The increased glucose variation could be considered as the positive glucose response under GLP-1 analogue in these patients with advanced type 2 DM.

In addition to mean glucose reduction, postprandial glucose improvement was disclosed in this study. Postprandial control was difficult in patients with type 2 DM due to the rice-based diets with high glycemic index in Taiwanese dietary habits. This rise and fall of postprandial glucose levels is mediated by the first-phase insulin response, in which large amounts of endogenous insulin are released in response to nutrient intake. In individuals with type 2 diabetes, the first-phase insulin response is severely diminished or absent, resulting in persistently elevated postprandial glucose throughout most of the day^12^. In current study, the improvement of *β*- cell function by GLP-1 analogue was the key point of the combination therapy via exenatide and intensive insulin therapy.

The Guideline for Management of Post-Meal Glucose in Diabetes of International Diabetes Federation (IDF) recommends that postmeal hyperglycaemia is harmful and should be addressed^13^. An independent and cumulative effect of postmeal hypertriglyceridaemia and hyperglycaemia related endothelial dysfunction might play an important role in the initiation of athrosclerosis^14^,^15^. The effect of exenatide in postprandial glucose control was still documented in these poorly controlled patients. Although the glucose variation was shown to be increased in the exenatide group, the impact of glucose variation on oxidative stress could be offset by an independent inhibitory effect of insulin therapy according to the reports by Monnier *et al*.^16^. It was also compatible with the results of no difference in 24-hour urine 8–iso-PGF_2α_ values between the exenatide and placebo groups. The cardiovascular biomarkers, adiponectin, hs-CRP and BNP, selected in this study were reported significantly correlated to the use of GLP-1 analogue in patients with type 2 DM^17^. There was a non-significant, but numerically, reduction in hs-CRP and BNP levels in GLP-1 analogue group. GLP-1 analogue could increase adiponectin mRNA level through the GLP-1 receptor and ameliorate insulin resistance via the protein kinase A pathway in 3T3-L1 adipocytes^18^. In this study, we demonstrate the role of GLP-1 analogue in human trial and provide the novel clinical implementation in these poorly controlled patients under advanced insulin therapy. The adiponectin could provide protection from endothelial dysfunction and prevent atherosclerotic formation. The improvement of adiponectin level in the current study also reflected the effect of GLP-1 analogue add-on intensive insulin therapy in the cardiovascular protection in addition to reduced insulin resistance. The negative correlation of the change of adiponectin level with the BMI and serum C-peptide level in the GLP-1 analogue group supported that even in patients with poorly controlled type 2 DM, the change of adiponectin by GLP-1 analogue was still moderate.

This is a study of hospitalized practice. All participants were hospitalized in a week. The pros of in-hospital designation are (1) the fixed daily food intake, the accuracy of blood glucose monitoring, and the regimen of medication are ensured and (2) the normalization of glucose levels by CSII can be achieved in a short period of time. Following the protocol from the pump therapy in our previous work^7^, we could just focus on reducing the basal infusion rate to prevent hypoglycemia and increasing bolus insulin dosage while fixed carbohydrate amount in meals. As a result, we can optimize the glucose levels with 3-day conditioning by CSII efficiently before further GLP-1 analogue add-on studies. The cons of this design are small number of participants and short of study period because of the expense and patient’s tepidity for hospitalization. The small size limits the usefulness of statistical analysis and this study lacks data on long-term A1C control for the GLP-1 analogue add-on CSII treatment in patients with poorly controlled type 2 DM.

In conclusion, the add-on of GLP-1 analogue to the intensive insulin therapy with CSII not only significantly showed further effect among glucose control but remarkably potentiated the *β*- cell function in patients with poorly controlled type 2 diabetes mellitus.

## Methods

### Patients

Patients with type 2 diabetes were enrolled into this study from 2011 to 2013. The inclusion criteria were: (a) age >20 years; (b) diabetes mellitus diagnosed >2 years; (c) A1C level at 8% to 12%; and (d) receiving premixed insulin twice daily with a total insulin daily dose >0.6 u/kg/day. The exclusion criteria were: (a) recent history of drug or alcohol abuse; (b) sensitivity to analogous products; (c) serious cardiovascular disorders; (d) participation in another clinical investigation study; (e) ongoing influenza, autoimmune disease or other metabolic disorders; and (f) pregnant or lactating women. This study was approved by the Institutional Review Board of Chang Gung Memorial Hospital and registered with ClinicalTrials.gov (NCT01473147). Written informed consent was obtained from each subject. All experiments were performed in accordance with the approved guidelines.

### Study protocol

All of the participants received a 6-day course of CSII intensive treatment during hospitalization (Supplement Figure 1). The finger-stick test was to examine pre-meal (AC) and 2-hour post meal (PC) glucose levels after three meals in addition to bedtime and nocturnal glucose levels for a total of 8 measurements a day. The glucose level was optimized (AC 70 – 140 mg/dl and PC 70 – 180 mg/dl) in the first 3 days, and the patients are assigned (through a computer-generated, random sequence) by a randomized, open-labled, parallel, placebo-controlled trial to combined therapy with exenatide 5 μg twice daily for 3 days or placebo by normal saline injection.

The 75 g oral glucose tolerance test (OGTT) was performed at baseline and at the end of study to assess the insulin sensitivity index and homeostasis model assessment-insulin resistance (HOMA-IR)^19^,^20^. We stopped pharmacological treatment for at least 12 hours (premixed insulin after the evening dose) before performing the 75 g OGTT at baseline. To eliminate the effect of ultra-short acting insulin, Aspart, in the use of CSII, the 75 g OGTT was performed 2 hours after CSII had been stopped (end of the study). To evaluate the effect of GLP-1 analogue, the 75 g OGTT at the end of the study was performed after injection of exenatide. The biomarkers, adiponectin, hsCRP, BNP and 24-hour urine 8–iso-PGF_2α_ levels were checked at baseline and endpoint.

### Continuous subcutaneous insulin infusion (CSII)

The insulin regimen was switched from pre-mixed insulin to CSII according to a previously described hospital-based protocol^7^,^21^. In brief, the pre-pump total daily dose of insulin was used as the starting dose of CSII. Half of the dose was infused continuously as the basal dose, and the other half was divided for each meal as the bolus dose. The basal insulin dose was then titrated as precisely as 0.1 U per hour to maintain the blood glucose targets in the range of 90–140 mg/dl from bedtime through the nocturnal period, and 70–140 mg/dl before each meal. The bolus insulin dose was titrated up or down carefully by 1 U for a fixed amount of carbohydrates to maintain the postprandial glucose range between 70–180 mg/dl. We found the setting of 50% of total daily dose as the basal insulin dose was usually overestimated in our patients. Therefore, we focused on reducing the basal infusion rate to prevent hypoglycemia and increased the bolus dosage for a fixed amount of carbohydrates during meals. Most of the patients received an adequate adjustment based on this 3-day titration protocol. At the end of the study, the switch in treatment of twice-daily or multiple-daily injections in CSII was equal to the divided total daily insulin dose or the total daily basal dose and respective pre-meal bolus dose according to a recommended protocol^7^. The medical team included diabetologists, educators, and dieticians, who were on call to manage any unexpected conditions during hospitalization.

### Laboratory Measurements

Plasma glucose was determined by the glucose dehydrogenase method (Wako Pure Chemical Industries, Ltd., Osaka, Japan) on LABOSPECT 008 Hitachi Automatic Analyzer (Hitachi, Ltd., Tokyo, Japan) with the intra-assay and inter-assay coefficients of variation 2.4% or less. A1C values were measured by Boronate Affinity & high-performance liquid chromatography (HPLC) (Premier Hb9210, Trinity Biotech, Kansas City, USA) with the intra-assay and inter-assay coefficients of variation 1.7% or less. C-peptide was measured by a solid-phase, two-site chemiluminescent immunometric assay (IMMULITE 2000 C-peptide assay, Siemens AG, Erlangen, Germany) with the intra-assay and inter-assay coefficients of variation 4.1% or less. Insulin levels were determined by chemiluminescent microparticle immunoassay (CMIA) (ARCHITECT Insulin assay, Abbott Laboratories, IL, USA). The within and between assay variations of insulin measurements were 2.7% or less. The hsCRP was determined by Latex agglutination turbidimetric immunoassay (QUALIGENT CRP, SEKISUI MEDICAL Co., Ltd., Tokyo, Japan) with the intra-assay and inter-assay coefficients of variation 3.3% or less. The BNP was measured by Alere Triage BNP Test (Alere Inc., MA, USA) with the Beckman Coulter Immunoassay System (Beckman Coulter, Inc., CA, USA). The intra- and interassay coefficient of variation was lower than 10%.

For the adiponectin assay, anti-human adiponectin monoclonal antibody (mAb) (R&D System, Minneapolis MN, USA) coated plates were reacted with 10 μl of standards or serum samples prediluted 100-folds and 90 μl anti-human adiponectin biotin-conjugated mAb (R&D System, Minneapolis, MN, USA) for 1 hour at 30 °C. For the second hour of incubation, AMDEX™ streptavidin-HRP (Amersham, GE, Piscataway NJ, USA) was added to amplify the signal. Absorbance was measured at 450 nm. The calibrators were recombinant proteins purchased from R&D Systems (R&D Systems, Minneapolis MN, USA) and were reconstituted according to the manufacture’s instructions. The standard solutions were serial diluted into five different concentrations as calibrators and 1% BSA buffer as the zero point. The five levels of calibrators were 1.6, 3.1, 6.5, 12.8 and 22.5 ug/mL.

Free 8-iso PGF_2α_is the most frequently measured F2 isoprostane in body fluids. In the present study, this isomer was measured using an enzyme immunoassay method (Cayman Chemical, Ann Arbor, Michigan, USA). The procedure was carried out according to manufacture’s manuals as previously described^22^. The 24-hour urinary excretion rate of 8-iso PGF_2α_was expressed as picogram per milligram urinary creatinine to minimize the consequences of inadequate urine collection and suppress the difference between men and women in urinary excretion rates.

### Calculation of *β* - cell function during 75 g OGTT

Area under the curve (AUC) for glucose and insulin during the OGTT were calculated by the trapezoid rule. Insulinogenic index was calculated as the ratio between incremental plasma insulin and glucose concentrations during the baseline and peak in the OGTT (Insulin_0–peak_/ Glucose_0–peak_). Total insulin secretion was calculated as the ratio between the incremental AUC of insulin and glucose during the OGTT (ΔInsulin AUC/ΔGlucose AUC). The Matsuda index was calculated for insulin sensitivity (10000/(fasting plasma glucose × fasting plasma insulin × mean OGTT glucose concentration × mean OGTT insulin concentration)^1/2^)^19^.

### Statistic analysis

The differences in the changes in continuous variables between 2 treatment groups were analyzed by using ANCOVA with baseline values were input as covariates. The paired Student’s *t*-test was used to compare differences before and after treatment in the same group. Differences in proportions were assessed using a chi-square test or Fisher’s exact test, as appropriate. Results were expressed as means ± standard error mean or percentage. Generalized estimating equation (GEE) analysis with robust standard error and exchangeable working correlation matrix was applied for repeated measurement. The level of statistical significance was set at a *p*-value of 0.05 or less. Statistical analyses were conducted with SAS (v9.3, SAS Institute, Cary, NC, USA).

## Additional Information

**How to cite this article**: Lin, C.-H. *et al*. Glucose Variability and *β*-Cell Response by GLP-1 Analogue added-on CSII for Patients with Poorly Controlled Type 2 Diabetes. *Sci. Rep*. **5**, 16968; doi: 10.1038/srep16968 (2015).

## Supplementary Material

Supplementary Information

## Figures and Tables

**Table 1:** Clinical characteristics of participants at baseline.

	Placebo added on CSII	GLP-1 analogue added on CSII	*p* - value
Number	21	30	
Gender (Male:Female) (%)	13 (62%) : 8 (38%)	16(53%) : 14(47%)	0.370
Age (years old)	53.5 ± 3.3	52.8 ± 2.4	0.871
BMI (kg/m^2^)	27.0 ± 1.1	28.9 ± 0.8	0.165
DM duration (years)	12.8 ± 1.9	11.6 ± 1.3	0.594
A1C % (mmol/mol)	10.6 ± 0.3 (92.1 ± 3.7)	10.5 ± 0.2 (91.3 ± 2.5)	0.859
C-peptide (ng/ml)	1.21 ± 0.27	1.68 ± 0.26	0.232

CSII: continuous subcutaneous insulin infusion.

BMI: body mass index.

Data was presented as mean ± SEM.

**Table 2:** Comparing unadjusted and adjusted mean glucose, SDPG and MAGE at end point between two groups.

Parameter	Placebo added on CSII	GLP-1 analogue added on CSII	*p*
Unadjusted (Mean ± SEM)			
Mean glucose (mg/dl)	152.35 ± 7.90	144.60 ± 4.22	0.393
SDPG (mg/dl)	44.35 ± 2.46	48.61 ± 3.26	0.302
MAGE (mg/dl)	86.71 ± 6.56	96.00 ± 7.07	0.366
^a^Adjusted (Mean ± SEM)			
Mean glucose (mg/dl)	153.36 ± 5.13	143.93 ± 4.15	0.167
SDPG (mg/dl)	41.49 ± 3.00	50.51 ± 2.43	0.027*
MAGE (mg/dl)	80.89 ± 7.53	99.87 ± 6.09	0.062

SDPG: standard deviation of plasma glucose.

MAGE: mean amplitude of glycemic excursions.

^a^It was adjusted for baseline values by ANCOVA and the following covariates: Age, Sex, BMI, DM duration and A1C

^*^p *<* 0.05 between two groups.

**Table 3:** Measures of glycemia and insulin secretion during 75-g OGTT before and after treatment.

Variable	Placebo added on CSII	GLP-1 analogue added on CSII	*p* value
Incremental Glucose AUC (mg /dl /h)			
Baseline	2296.00 ± 138.41	2328.67 ± 83.62	0.831
Endpoint	2156.80 ± 94.71	1670.83 ± 90.28	0.001
Change from baseline	−139.20 ± 125.24	−657.83 ± 103.95	0.001*
Incremental Insulin AUC (mU/ml/h)			
Baseline	124.70 ± 22.70	280.95 ± 103.17	0.229
Endpoint	105.37 ± 23.23	301.09 ± 127.02	0.219
Change from baseline	−19.33 ± 24.94	20.14 ± 35.98	0.358
Insulin_0–peak_/Glucose_0–peak_ (mU/mg)			
Baseline	0.03 ± 0.01	0.12 ± 0.07	0.250
Endpoint	0.03 ± 0.02	0.22 ± 0.07	0.054
Change from baseline	0.01 ± 0.01	0.10 ± 0.03	0.007*
Matsuda index			
Baseline	4.46 ± 1.17	4.43 ± 1.03	0.988
Endpoint	7.90 ± 2.31	5.03 ± 0.80	0.250
Change from baseline	3.45 ± 2.34	0.60 ± 1.04	0.253
ΔInsulin AUC/ΔGlucose AUC (mU/mg)			
Baseline	0.06 ± 0.01	0.14 ± 0.05	0.243
Endpoint	0.05 ± 0.01	0.21 ± 0.10	0.185
Change from baseline	−0.01 ± 0.01	0.07 ± 0.05	0.088

Data presented as mean ± standard error mean.

Δincremental.

^*^p < 0.05 by robust standard error and exchangeable working correlation matrix of generalized estimating.

**Table 4:** Comparisons of biomarkers between two groups.

Variable	Placebo added on CSII	GLP-1 analogue group added on CSII	*p* value
Adiponecitn (μg/mL)			
Baseline	14.96 ± 3.08	9.39 ± 1.69	0.093
Endpoint	13.26 ± 3.75	9.78 ± 1.81	0.275
Change from baseline	−1.62 ± 0.97	0.39 ± 0.32	0.045*
hsCRP (mg/L)			
Baseline	2.52 ± 0.51	3.90 ± 0.84	0.146
Endpoint	1.88 ± 0.37	2.87 ± 0.45	0.116
Change from baseline	−0.61 ± 0.46	−1.03 ± 0.81	0.508
BNP (pg/mL)			
Baseline	28.57 ± 9.91	16.47 ± 3.48	0.261
Endpoint	19.96 ± 6.62	7.80 ± 1.02	0.084
Change from baseline	−8.21 ± 4.65	−8.68 ± 3.34	0.991
8–iso-PGF_2α_ (pg/mg /creatinine)			
Baseline	1265.63 ± 167.15	1102.90 ± 94.50	0.367
Endpoint	1070.29 ± 126.91	1055.64 ± 103.11	0.929
Change from baseline	−186.04 ± 84.45	−47.27 ± 58.39	0.152

Data presented as mean ± standard error mean.

^*^p < 0.05 by robust standard error and exchangeable working correlation matrix of generalized estimating equation (GEE) analysis.

**Table 5:** Correlation of changes of adiponectin level, insulinogenic index, and SDPG with the baseline characteristics in GLP-1 analogue group.

Parameter	Adiponectin change (μg/mL)		Insulinogenic index change (mU/mg)		SDPG change (mg/dL)	
	Correlation coefficients	*p* value	Correlation coefficients	*p* value	Correlation coefficients	*p* value
Age (years old)	−0.254	0.176	−0.171	0.366	−0.074	0.699
BMI (kg/m^2^)	−0.403*	0.027	−0.165	0.385	0.197	0.298
DM duration (years)	−0.315	0.090	−0.105	0.581	0.007	0.969
A1C %	0.169	0.373	−0.032	0.865	−0.002	0.992
C-peptide (ng/mL)	−0.426*	0.019	−0.182	0.337	0.129	0.497

SDPG: standard deviation of plasma glucose.

^*^Correlation is significant at the 0.05 level (2-tailed).
