# Supplementary material for: Glucose Variability and β- Cell Response by GLP-1 Analogue added-on CSII for Patients with Poorly Controlled Type 2 Diabetes
Source: Sci Rep. 2015 Nov 26;5:16968. doi: 10.1038/srep16968 (PMC4660278; doi:10.1038/srep16968)
Supplement: Supplementary Information [file srep16968-s1.pdf]

Glucose Variability and β-Cell Response by GLP-1 Analogue added-on CSII for Patients with Poorly Controlled Type 2 Diabetes

Chia-Hung Lin[1,2], Sheng-Hwu Hsieh[1], Jui-Hung Sun[1], Jir-Shiong Tsai[3], Yu-Yao Huang[1]

Supplement Figure legends:

Supplement - Figure 1. The study design of radomized, open lable trial in GLP-1 analogue added-on CSII

Supplement - Figure 2. The beta cell response in 75 g OGTT_glucose change

△ G (0-120) : The glucose change from 0 minute to 120 minutes

Supplement - Figure 3. The beta cell response in 75 g OGTT_insulin change

△ I (0-120) : The insulin increment from 0 minute to 120 minutes

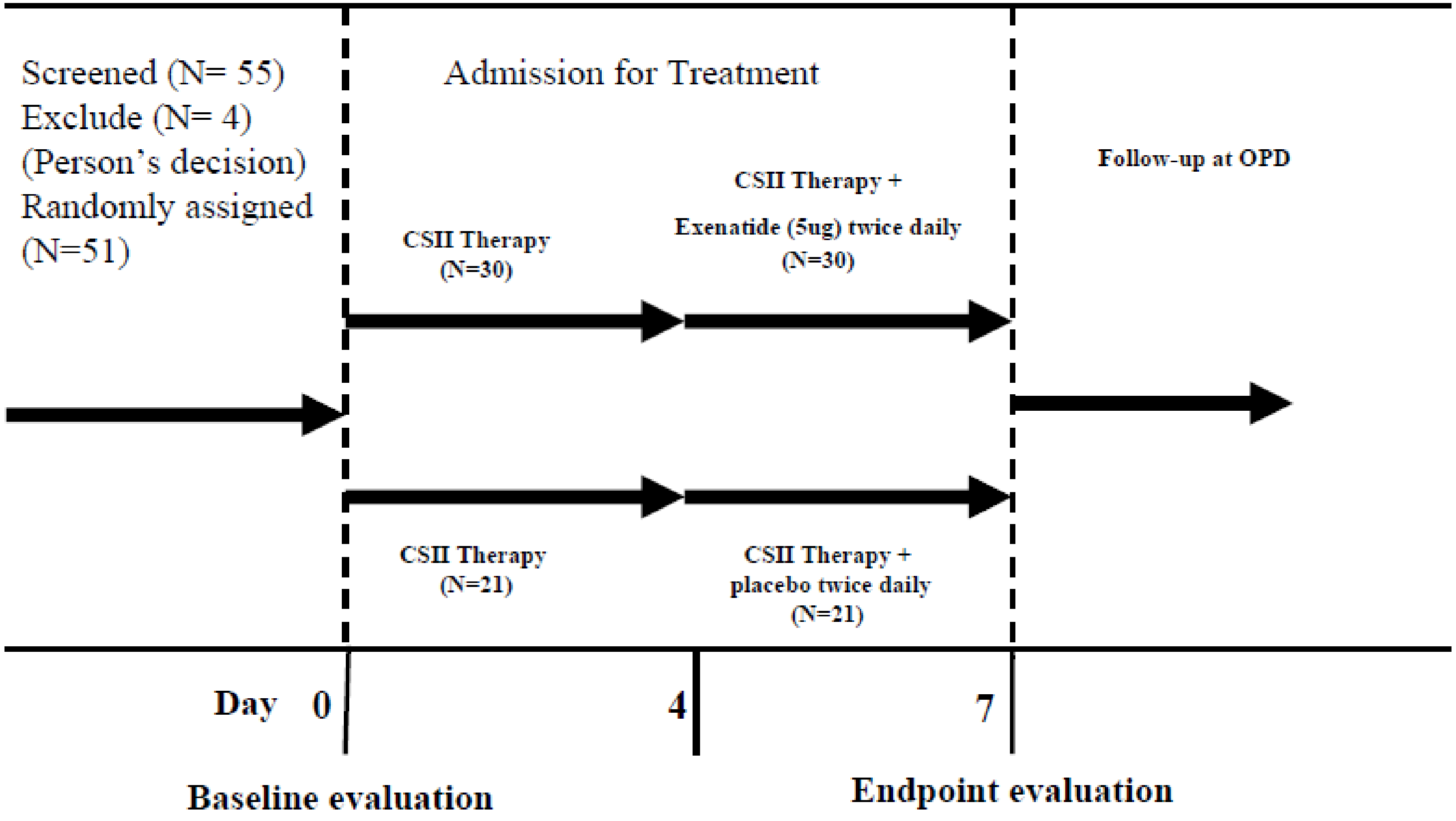

Supplement - Figure 1

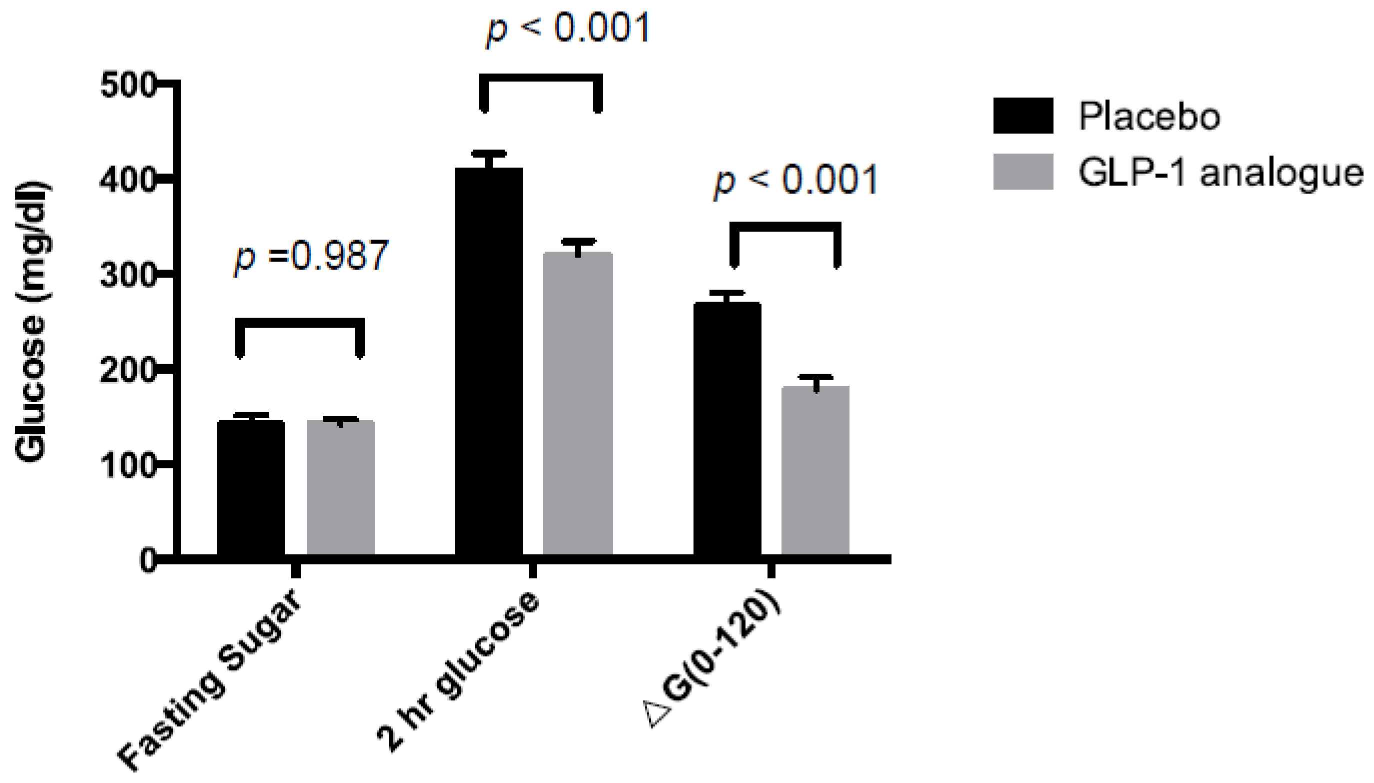

Supplement - Figure 2

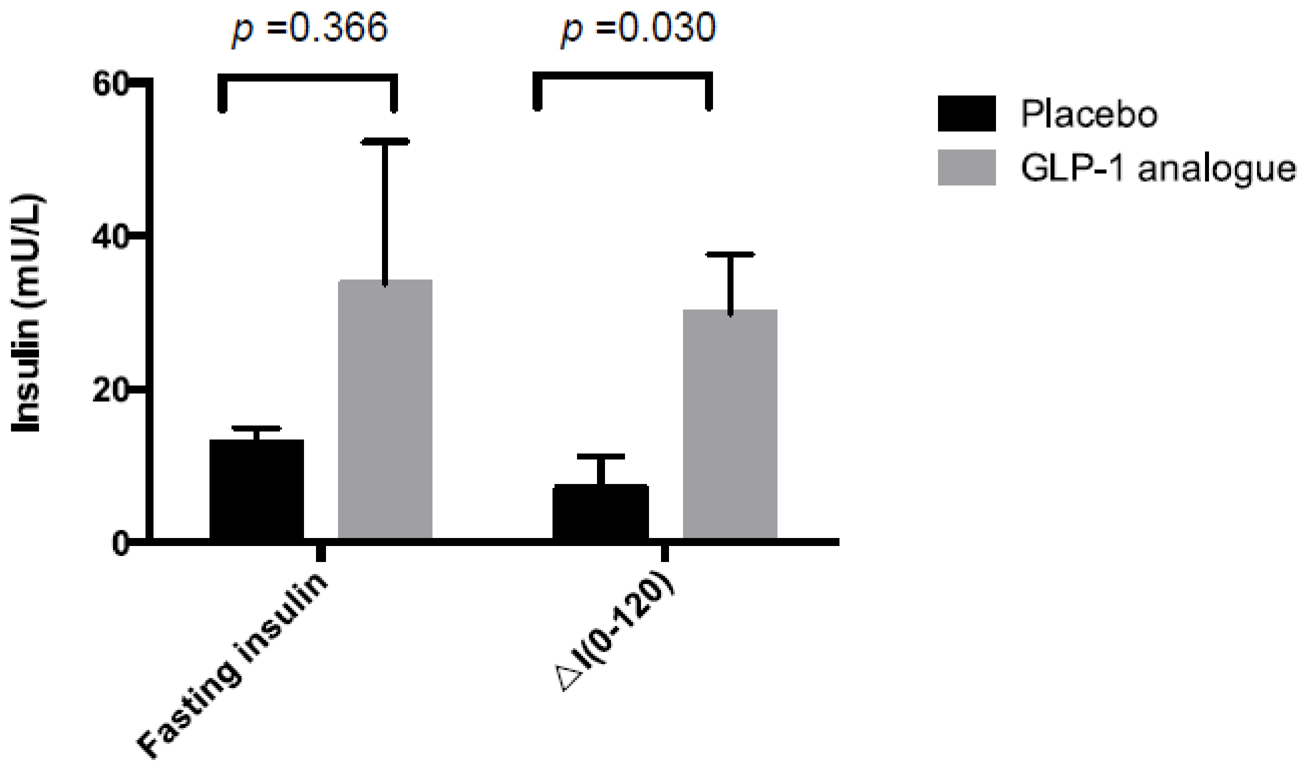

Supplement - Figure 3

Supplement - Table 1. Insulin dose between two groups

Parameter	Placebo added on CSII	GLP-1 analogue added on CSII	*p*
TDD (U/kg)_baseline	0.83 ± 0.07	0.88 ± 0.04	0.511
TDD (U/kg)_endpoint	0.82 ± 0.06	0.85 ± 0.04	0.716
Basal (U/kg)_baseline	0.37 ± 0.03	0.41 ± 0.02	0.497
Basal (U/kg)_endpoint	0.36 ± 0.03	0.41 ± 0.02	0.224
Bolus (U/kg)_baseline	0.46 ± 0.04	0.47 ± 0.02	0.824
Bolus (U/kg)_endpoint	0.47 ± 0.03	0.44 ± 0.02	0.605

TDD: total daily dose

Basal: Basal insulin

Bolus: Bolus insulin

Supplement - Table 2. Number of Participants Reporting Adverse Events

Variables	Placebo added on CSII (n=21)	GLP-1 analogue added on CSII (n=30)	*p*
Patient with SAE	0	0	
Death	0	0	
Abdominal fullness	0 (0)	11 (36.7)	0.001
Diarrhea	0 (0)	1 (3.3)	0.588
Nausea	0 (0)	4 (13.3)	0.110
Vomiting	0 (0)	4 (13.3)	0.110
Dizziness	0 (0)	2 (6.7)	0.341
Hypoglycemia			
Over all	9 (43)	12 (40)	0.838
Nocturnal	2 (9.5)	3 (10)	1.000
Severe	0 (0)	0 (0)	1.000

Data presented with number (%)

Supplement - Table 3 The comparisons of the established index of insulin secretion (C-peptide / glucose [ng . ml^{-1} . mg^{-1} . dl]) during 75 g OGTT

	Placebo added on CSII (N = 21)		GLP-1 analogue added on CSII (N = 30)		
	Baseline	End Point	Baseline	End Point	*p* – value*
Fasting	0.0075±0.0020	0.0105±0.0038	0.0080±0.0012	0.0137±0.0022	0.389
Fasting Mean change	0.0030±0.0027		0.0057±0.0017		
30 minutes	0.0068±0.0017	0.0075±0.0018	0.0080±0.0010	0.0147±0.0021	< 0.001
30 minutes Mean change	0.0007±0.0007		0.0068±0.0014		
60 minutes	0.0069±0.0017	0.0082±0.0020	0.0084±0.0010	0.0161±0.0023	0.001
60 minutes Mean change	0.0013±0.0011		0.0077±0.0017		
90 minutes	0.0074±0.0018	0.0084±0.0022	0.0086±0.0010	0.0190±0.0024	< 0.001
90 minutes Mean change	0.0009±0.0012		0.0104±0.0020		
120 minutes	0.0077±0.0019	0.0102±0.0026	0.0095±0.0011	0.0220±0.0028	< 0.001
120 minutes Mean change	0.0026±0.0010		0.0125±0.0023		

Data shown as mean ± SEM

*: *p* value derived by robust standard error and exchangeable working correlation matrix of generalized estimating equation (GEE) analysis
